# Prevalence of infectious keratitis in Central China

**DOI:** 10.1186/1471-2415-14-43

**Published:** 2014-04-02

**Authors:** Jin Cao, Yanning Yang, Wanju Yang, Ruoxi Wu, Xuan Xiao, Jing Yuan, Yiqiao Xing, Xiaodong Tan

**Affiliations:** 1 Department of Ophthalmology, Wuhan University, Renmin Hospital, Wuhan 430060, Hubei Province, People’s Republic of China; 2 School of Public Health, Wuhan University, Wuhan 430060, Hubei Province, People’s Republic of China

**Keywords:** Infectious keratitis, Corneal diseases, Corneal blindness, Visual ability

## Abstract

**Background:**

The baseline data pertaining to the national epidemiological survey of infectious keratitis remain scarce in China, and currently there is no corneal blindness control strategy developed by the nation.

**Methods:**

Geographically defined cluster sampling was used to randomly select a cross-section of residents from representative urban and rural populations in Hubei Province. Participants were selected from village registers, followed by door-to-door household visits. The assessment items included a structured interview, visual acuity testing, external eye examination, and anterior segment examination using slit lamp. Causes and sequelae of corneal disease were identified according to uniform customized protocol.

**Results:**

The prevalence of presenting corneal diseases was 0.8% (211/26 305), while the prevalence of infectious keratitis was 0.148% (39/26 305). The prevalences of viral, bacterial, and fungal keratitis were 0.065, 0.068, and 0.015%, respectively. There were no significant differences found between the prevalences of viral (accounting for 43.6%) and bacterial (accounting for 46.2%) corneal ulcers. cases of *Acanthamoeba* keratitis were not found. Infectious keratitis was the leading cause of corneal blindness (85.7%), and the prevalence of blindness in at least one eye resulting from infected corneas was 0.091% (95% CI: 0.067-0.127%).

**Conclusions:**

Viral and bacterial mechanisms constitute the most important risk factors for infectious corneal ulcers in Central China. To reduce the rate and severity of infectious keratitis, he public health care policy should be focused on designing cost-effective strategies and operational programs for the prevention and prompt treatment of infectious corneal ulcers.

## Background

Infectious keratitis is still one of the main causes of corneal blindness and visual Disability [1]. In a study by Bourne et al find that leading causes worldwide in 2010 for blindness were cataract 33%, uncorrected refractive error 21%, and macular degeneration 7%, and for moderate and severe vision impairment were uncorrected refractive error 53%, cataract 18%, and macular degeneration 3% [2]. Causes of blindness varied substantially by region. Corneal diseases are the most common causes of blindness in countries with less developed economies, second only to cataracts in overall importance [1,3,4]. The etiology of corneal blindness is complicated and encompasses a wide variety of infectious and inflammatory eye diseases that result in severe outcomes. In addition, the prevalence of corneal disease varies by country and population.

In developing countries, most patients with infectious keratitis do not receive medical care due to discounting the seriousness of their condition or poverty. In addition, the lack of effective drugs, essential checking and operating equipment, and well-trained medical care personnel, as well as a lack of basic medical insurance systems and a shortage of cornea grafts, result in severe outcomes.

It is clear that the epidemiology of corneal blindness is diverse and highly dependent on the ocular diseases that are endemic to each geographical area. Through the perseverance of public health programs, the morbidity of corneal diseases due to *Chlamydia trachomatis*, onchocerciasis, and leprosy have been controlled relatively stable [1]. Research efforts throughout the world are now focused on studying other causes of corneal blindness, including ocular trauma, corneal ulceration, and complications from ophthalmic drug interactions. Currently, infectious keratitis is mainly caused by viruses, fungi, bacteria, and *Acanthamoeba*.

China is an agricultural country with a population of 1.3 billion people, which accounts for 15% of the world’s population. With the global figure of trauma and corneal ulcers are responsible for around 1.5-2.0 million cases of corneal blindness [1,5], China is estimated to have the largest number of blind people globally [4]. Despite the great efforts of the Chinese government to address the universal healthcare circumstance, poor access and high costs remain the 2 major problems in China’s health system. China’s First National Sample Survey of Disabled Persons suggested that corneal disease as a major cause of blindness and low vision ranks second only to cataract in china, and the prevalence of corneal blindness and low vision was 11.5/10000, accounting for approximately one-sixth of the cases of blindness in China [6].

Common tools used to develop strategies for controlling infectious corneal blindness are epidemiological surveys. However, relevant data from epidemiological surveys of infectious keratitis in China remain scarce. Funded by grants from the Chinese Academy of Engineering, the Shandong Eye Institute initiated a program called “the Social Harm and Intervention Strategies of Infectious Keratitis in China.” This program constitutes the first national multicenter epidemiological survey of infectious keratitis.

According to the “sixth national census in 2010 Communiqué on Major Data of Hubei,” the resident population of China is 57,237,740. In our study, a random sampling of 26 305 people (0.046% of the total population) consisted of 56% (14 728 individuals) rural and 44% (11 577 individuals) urban populations. Hubei Province is located along the Yangtze River, the main artery of communication between North and South China, north of the Dongting Lake. This region has a subtropical humid monsoon climate and an annual average temperature of 15.9-16.6°C. Transportation is possible by air, water, and land; therefore, the distribution of disease species in a particular area reflects the national average species distribution. It is important for ophthalmologists to obtain a more clear and concise understanding of the epidemiology of infectious keratitis. The aim of this study was to understand the prevalence and risk factors of infectious keratitis in the Hubei area and to then calculate the prevalence of infectious keratitis in Central China as a means of providing a foundation for comprehensive prevention in the community and the curing of corneal disease.

## Methods

### Study areas

This study utilized a multi-stage stratified cluster random sampling technique. The random, rural area sample group consisted of individuals in Wuhan City, which contains Zhonghua Road, Ziyang Road, and Shouyi Road, and the urban area sample group consisted of individuals from the Taihu farms in Jinzhou City. The streets (townships) in all neighborhoods (villages) were extracted by registration number and geographical location to form random groups, each containing approximately 1000 people (all ages). Each group was sorted by simple random sampling.

The site investigation occurred from January 1 to August 31, 2010. We verified registration names, genders, ages, and education levels from the census data provided by the Residents Committee by conducting door-to-door household visits.

We have a special checkpoint in Community Neighborhood Committee or villages, and The investigation team was composed of specialized doctors, nurses, and community (village) staff, all of whom had received training before administration of the survey. The training included instruction on how to perform a standard investigation, visual acuity assessment, and diagnosis, as well as on how to administer the survey. We performed a preliminary survey, the results of which are not included in this paper. Verbal consents were obtained from all participants prior to the examination. For those minors aged less than 18 years, the survey was approved by their guardians.

The site investigations consisted of 2 stages.two-stage investigations at the same location. Those who did not come to the examinations were re-visited and encouraged to participate in the study, or we detected them in their houses using portable equipments include a visual acuity chart, portable slit lamp and portable flashlight. The response rate was kept more than 80% in each cluster. In the first stage, the standard logarithmic visual acuity chart was used to assess distance vision, the anterior segment was assessed via a slit-lamp evaluation, and the corneal epidemiological survey form was completed. The inclusion criteria for enrollment in the next stage of the study included the following: the presence of corneal lesions (eg, corneal ulcers), infiltration, edema, scarring, opacity, neovascularization, degeneration, pterygium, keratoplasty, foreign bodies (arcus senilis not included), or atrophy caused by corneal disease.

In the second stage, we obtained a detailed history of systemic disease, systemic therapy, ocular trauma, contact lens usage, and eye disease diagnosis and treatment. We also performed a slit-lamp examination and took photographs of corneal lesions. If we found corneal infiltrates/ulcers with stromal invasion > 1 mm^2^ (with or without epithelial defects) or corneal ulcers > 1 mm^2^ (with or without hypopyon), infectious keratitis was suspected. All suspected cases were invited to clinics and performed a corneal scraping and conducted a pathological examination. The information on and diagnosis of each subject in the second stage were recorded in uniform medical records. Patients who were found to have infectious keratitis were recommended to complete the socioeconomic survey.

### Definitions and standards

1. Blind and low vision: According to the WHO, a visual acuity of < 20/60 of the better eye in daily life is defined as visual impairment, including blindness (< 20/400) and low vision (presenting visual acuity ≥ 20/400, < 20/60).

2. Corneal blindness: Visual acuity of < 20/400 in one eye resulting from corneal disease is defined as corneal blindness.

3. Diagnostic criteria for infectious keratitis:

#### Active inflammation

(1) Fungal and amoebic keratitis is diagnosed using an etiological test consisting of a smear, culture, confocal microscopy, and pathological examination, and a diagnosis is made if at least one of these tests is positive.

(2) Viral keratitis is diagnosed according to a history of recurrent, typical signs, and symptoms.

(3) Bacterial keratitis is diagnosed according to smear and culture results. If the culture test is negative but antibacterial treatment is effective, a diagnosis that excludes fungal, amoebic, viral, and noninfectious keratitis can be made immediately.

(4) The prevalence of corneal disease/infectious keratitis is a statistic that includes subjects who either have a history of this disease or currently have this disease.

### Statistical analysis

Ethical approval for the study was granted by the Ethics Committee of the Shandong Eye Institute, Qingdao, China. Before the examination, we obtained the oral consent of each subject or permission from their guardian (for subjects under 18 years of age). The raw data were entered into an Excel table. After troubleshooting, we transferred these data into the SPSS 17.0 database; data analysis was performed using the SPSS 17.0 software package (SPSS Inc., Chicago, IL). The χ^2^ test was performed to assess the differences in the prevalence of diseases significant judgments; *P* < 0.05 indicated that the differences were statistically significant.

## Results

In the Hubei regions, A total of 27,312 subjects were recruited and 26,305 individuals finished the examine and test with a response rate of 96.3%. 11 577 in the urban residential cohort, and 14 728 in the rural residential cohort. The average age was 41.8 ± 19.4 years (range: 0.0 to 98.0). The prevalence of corneal disease presentation was 0.8% (211/26 305), and the prevalence of infectious keratitis was 0.148% (39/26 305) (Table 1) and was the leading cause of corneal blindness (85.7%) (Table 2). The prevalences of viral keratitis, bacterial keratitis, and fungal keratitis were 0.065, 0.068, and 0.015%, respectively (Table 3). Distribution of corneal diseases. Numbers of pterygium, infectious keratitis, traumatic scar and unknown cause scars in rural and urban respectively is 126, 28, 10, 11 and 13, 11, 3, 9. A large proportion (65.9%) of the 211 individuals with corneal disease in our investigation had a pterygium lesion (Table 4).

**Table 1:** The prevalence of corneal disease and infectious keratitis

	**Corneal disease**	**Infectious keratitis**
Rural area No.	175	28
Urban area No.	36	11
Total No.	211	39
Prevalence %	0.8%	0.148%

The prevalence of corneal disease was 0.8%, Infectious keratitis was diagnosed in 39 eyes (0.148%) (Table 1) and was the leading cause of corneal blindness.

**Table 2:** Distribution of causes of corneal blindness

**Corneal diseases**	**Blindness in one eye**	**Blindness in two eyes**	**Blindness in at least one eye**
	**No. (%)**	**No. (%)**	**No. (%)**
Infectious corneal diseases	23 (82.1)	1 (3.6)	24 (85.7)
Pterygium-related diseases	1 (3.6)	0 (0)	1 (3.6)
Traumatic corneal scar	1 (3.6)	0 (0)	1 (3.6)
Unknown corneal opacity	1 (3.6)	1 (3.6)	2 (7.1)
Total	26 (92.9)	2 (7.1)	28 (100.0)

The infectious corneal disease ranked first among causes of corneal blindness, accounting for 85.7%.

**Table 3:** Infectious keratitis categories

	**No. of examined participants**	**VK**	**BK**	**FK**	**Total**
		**No. (%)**	**No. (%)**	**No. (%)**	**No. (%)**
Rural areas	14 728	9 (0.061)	15 (0.101)	4 (0.027)	28 (0.190)
Urban areas	11 577	8 (0.069)	3 (0.026)	0 (0.000)	11 (0.095)
Total	26 305	17 (0.065)	18 (0.068)	4 (0.015)	39 (0.148)

The distribution of infectious keratitis subtypes was as follows: 17/39 (43.6%) viral keratitis, 18/39 (46.2%) bacterial keratitis, and 4/39 (10.2%) fungal keratitis. The prevalences of viral keratitis, bacterial keratitis, and fungal keratitis were 0.065, 0.068, and 0.015%, respectively.

VK, viral keratitis; BK, bacterial keratitis; FK, fungal keratitis.

**Table 4:** Numbers of different types of corneal disease

**Corneal disease**	**Rural area**	**Urban area**	**Total**	**Prevalence**
	**No.**	**No.**	**No.**	**%**
Pterygium	126	13	139	65.9%
Infectious keratitis	28	11	39	18.5%
Traumatic scar	10	3	13	6.1%
Unknown cause scars	11	9	20	9.5%

Distribution of corneal diseases. Numbers of pterygium, infectious keratitis, traumatic scar and unknown cause scars in rural and urban respectively is 126, 28, 10, 11 and 13, .11, 3, 9. A large proportion (65.9%) of the 211 individuals with corneal disease in our investigation had a pterygium lesion.

The age, gender, education and rural - urban disparities distributions of each cohort are detailed in Table 5. The distribution of infectious keratitis subtypes was as follows: 17/39 (43.6%) viral keratitis, 18/39 (46.2%) bacterial keratitis, and 4/39 (10.2%) fungal keratitis (Table 3). The infections in 16 subjects were due to herpes simplex keratitis, while adenovirus was responsible for only 1 case of infectious keratitis. Bacterial keratitis accounted for the largest portion of cases, and were found in 46.2% of all infect cases. cases of Acanthamoeba keratitis were not found. The prevalence rate of BK, VK and FK in rural and urban regions respectively is 0.101%, 0.061%, 0.027% and 0.026%, .069%, 0.000%. In rural areas, with bacterial corneal ulcers being the most common cause 15/28(accounting for 53.6%), followed by viral 9/28(32.1%) and fungal 4/28(14.3%) keratitis. However, in urban areas, viral corneal keratitis was by far the most common cause 8/11(accounting for 72.7%), followed by bacterial keratitis 3/11(27.3%) (Table 3).

**Table 5:** Univariate analysis of the prevalence of infectious corneal diseases

**Characteristic**	**No. of examined participants**	**Corneal disease**	**χ**^**2**^	**Infectious keratitis**	**χ**^**2**^
		**No. (%, 95% CI)**	***P***	**No. (%, 95% CI)**	***P***
**Gender**			28.794		2.765
			<0.001		0.096
Male	12 942	65 (0.50, 0.38-0.62)		14 (0.11, 0.05-0.17)	
Female	13 363	146 (1.09, 0.91-1.27)		25 (0.19, 0.12-0.26)	
**Age (years)**	3 cases missing		281.000		48.333
			<0.001		<0.001
≤14	2029	1 (0.05, 0.04-0.06)		0	
15-59	19 196	74 (0.39, 0.30-0.48 )		12 (0.06, 0.03-0.09)	
≥60	5077	136 (2.68, 2.24-3.12 )		27 (0.53, 0.33-0.73)	
**Education**	11 cases missing		235.300		22.485
			<0.001		<0.001
None	2486	75 (3.02, 2.35-3.69)		13 (0.52, 0.24-0.80)	
Primary	6388	86 (1.35, 1.21-1.49)		12 (0.19, 0.08-0.30)	
High school	12 230	37 (0.30, 0.20-0.40)		8 (0.07, 0.02-0.12)	
College	3141	9 (0.29, 0.10-0.48)		4 (0.13, 0.01-0.25 )	
Postgraduate	2049	4 (0.20, 0.01-0.39)		2 (0.10, -0.04-0.24 )	
**Area**			62.691		3.960
			<0.001		0.047
Urban area	11 577	36 (0.31, 0.21-0.41)		11 (0.10, 0.04-016)	
Rural area	14 728	175 (1.19,1.01-1.37)		28 (0.19, 0.12-0.26)	

Corneal disease presented in higher prevalence in the female gender, rural areas and those receiving less education, and the prevalence increased with age.

Corneal disease was more common in women (χ^2^ = 28.794, *P* < 0.001), older age groups (≥ 60 years of age) (χ^2^ = 281.000, *P* < 0.001) and individuals with lower levels of education (χ^2^ = 235.300, *P* < 0.001) in the rural population (χ^2^ = 62.691, *P* < 0.001). The survey indicate that infectious keratitis presented in higher prevalence in the patients who live in rural areas and those receiving less education, and the prevalence increased with age (Table 5). The prevalence of infectious corneal blindness differed with respect to residence location (urban vs. rural) (χ^2^ = 30.613, *P* < 0.001), gender (χ^2^ = 2.765, *P* = 0.096), age (χ^2^ = 48.333, *P* < 0.001), and educational level (χ^2^ = 22.485, *P* < 0.001) (Table 5). Infectious keratitis was the leading cause of corneal blindness (85.7%) (Table 2), and the prevalence of blindness in at least one eye resulting from infected corneas was 0.091% (95% CI: 0.067-0.127%) (Table 6).

**Table 6:** The prevalence of corneal and infectious corneal diseases leading to corneal blindness

	**Blindness in one eye no.**	**Blindness in two eyes no.**	**Blindness in at least one eye no.**
	**(prevalence (%), 95% CI)**	**(prevalence (%), 95% CI)**	**(prevalence (%), 95% CI)**
Corneal diseases	26	2	28
	(0.099, 0.061-0.137)	(0.008, -0.003-0.019)	(0.106, 0.073-0.145)
Infectious corneal diseases	23	1	24
	(0.087, 0.051-0.123)	(0.004, -0.004-0.012)	(0.091, 0.067-0.127)

The prevalence of blindness in at least one eye resulting from infected corneas was 0.091% (95% CI: 0.067-0.127%).

## Discussion

Corneal disease is a major cause of blindness worldwide. However, the prevalence and causes of corneal blindness vary between different countries, geographic regions, and ethnic groups [7]. For more effective prevention and treatment of keratitis, it is important for ophthalmologists to be aware of regional epidemiological features, risk factors, and etiological data concerning this disease. This survey was designed to investigate the cause-specific, population-weighted prevalence of infectious keratitis in Central China.

Corneal keratitis has become an important cause of visual loss in many developing countries, where a large portion of the population consists of farm workers. this study is the first nationwide effort to conduct population-based, multicenter epidemiological investigation, trying to understand the burden, reasons, and demographic characteristics of corneal blindness.

This study produced interesting and useful data on the epidemiology of infectious keratitis. A large proportion of the 211 individuals with corneal disease in our investigation had a pterygium lesion, which is more likely strongly related to spending longer periods of time working outdoors and ocular sun exposure [8,9] , but this condition is not the main cause of blindness in rural Hubei (Table 2).

The age, gender, and education distributions of each cohort correspond to the population distributions of visual impairment reported by the WHO [7,10]. infectious keratitis presented in higher prevalence in the rural areas and those receiving less education, and the prevalence increased with age. Females, individuals living in rural areas, and individuals with lower education levels suffered from corneal diseases and blindness with a higher prevalence that increased with age compared with the male groups. The rate of blindness in at least one eye due to corneal disease was 0.106% in Hubei, while the rates in other regions ranged from 0.037% in Qinghai to 0.666% in Ningxia. If these data are extrapolated to the total population of Hubei, approximately 45.79 million individuals suffer from corneal disease, approximately 8.4712 million individuals of whom suffer from infective keratitis.

The prevalence of infective keratitis was higher in rural than urban areas. Most likely as a result of most patients’ For financial reasons to choose self-medication, most patients delayed seeking professional medical care [11]. There were no significant differences found between the prevalences of viral (accounting for 0.065%) and bacterial (accounting for 0.068%) corneal ulcers among the Hubei participants (Table 3). But there are urban and rural difference on the the prevalence rate of BK, and FK. The prevalence rate of bacterial keratitis in rural regions was approximately 4 times as high as the rate in urban regions. Infectious corneal keratitis, including fungal and *Acanthamoeba* keratitis, played an almost negligible role in the etiology of visual impairment in Central China. Thus, bacterial keratitis is the main cause of visual impairment in rural areas, especially in Central China.

These results partially contrast with those of other concurrent population-based studies in other areas. In China, the prevalence of blindness in at least one eye in rural areas is 3 times the prevalence of that in urban areas. There are several explanations for this prevalence discrepancy between urban and rural areas. First, 54% of the population in Hubei continues to live in rural areas [12], and a higher prevalence of blindness is associated with a lower socioeconomic status [13]. Second, the prevalence of visual impairment and blindness is comparatively high in rural populations due to the limited accessibility and affordability of eye care services for these individuals [14,15]. Third, in this survey, it is possible that the difference in prevalence between the rural and urban areas is primarily a reflection of differences in daily jobs, lifelong habits and the living environment. Fourth, recurrent attacks of bacterial keratitis are particularly frequent in the presence of constitutional factors, such as dry eye syndrome or eyelid abnormalities [16]. These types of data are becoming increasingly important, as they help us to realize the differences between urban and rural patients with infectious keratitis and to be able to offer appropriate care and treatment to both groups. The actual prevalence of infectious keratitis is not known. Keratitis caused by HSV is the most common cause of cornea-derived blindness in developed nations [17], The incidence of corneal ulceration per 100 000 individuals per year is estimated to vary from 6.3 in Hong Kong [18] and 11 in the United States [19] to 478 in southern England [20] and 799 in Nepal [21]. A report from India showed that 44% of all central corneal ulcers were caused by fungi [22].

Corneal disease prevention programs targeting rural populations should pay particular attention to areas in which individuals have limited access to and are unable to afford eye care services. Thus, particularly in Central China, Chinese governments and health institutions should initiate programs directed toward patient education, prevention, and prompt treatment of infectious corneal ulcers. More importantly, patients with symptoms of corneal ulcers should have prompt access to medical care and should avoid using over-the-counter medications, particularly those that contain corticosteroids.

## Conclusions

Numerous behavioral and structural components pertaining to eye care delivery, including attitudes and health care availability, accessibility, and affordability, affect visual impairment/blindness rates in rural and urban areas. Infectious keratitis was mainly found in individuals living under poor economic conditions; however, the expense of the treatment is more than these individuals can afford. Thus, particularly in Central China, the public health care policy should be focused on designing cost-effective strategies and operational programs for the prevention and prompt treatment of infectious corneal ulcers. The government should provide proper financial provisions for infectious keratitis patients and increase the reimbursement of treatment through medical insurance coverage. Thus, we hope that more patients will have the opportunity to regain their sight, thereby mitigating their physical suffering and mental distress.

## Competing interests

The authors declare that they have no competing interests.

## Authors' contributions

All authors made contribution to the manuscript. CJ carried out investigation analysis and summarize the whole survey, participated in drafted the manuscript. YNN conceived of the study, and participated in its design and coordination and helped to draft the manuscript. YWJ participated in the design of the study and performed the statistical analysis. XX contributed to design, interpreting data, WRX and YJ execute this investigation and to coordinate the work of the team. XYQ contributed to design, interpreting data, and critically revised the manuscript. TXD performed the statistical analysis. All authors read and approved the final manuscript.

## Pre-publication history

The pre-publication history for this paper can be accessed here:

http://www.biomedcentral.com/1471-2415/14/43/prepub
